# Genome-Scale Transcriptome Analysis of the Alpine “Glasshouse” Plant *Rheum nobile* (Polygonaceae) with Special Translucent Bracts

**DOI:** 10.1371/journal.pone.0110712

**Published:** 2014-10-24

**Authors:** Lizhong Wang, Haihong Zhou, Jin Han, Richard I. Milne, Mingyu Wang, Bingbing Liu

**Affiliations:** 1 State Key Laboratory of Grassland Agro-ecosystem, School of Life Science, Lanzhou University, Lanzhou, Gansu, China; 2 MOE Key Laboratory of Cell Activities and Stress Adaptations, School of Life Sciences, Lanzhou University, Lanzhou, Gansu, China; 3 Institute of Molecular Plant Sciences, School of Biological Sciences, University of Edinburgh, Edinburgh, United Kingdom; 4 Royal Botanic Garden Edinburgh, Edinburgh, Scotland, United Kingdom; Nanjing Forestry University, China

## Abstract

**Background:**

*Rheum nobile* is an alpine plant with translucent bracts concealing the inflorescence which produce a “glasshouse” effect promoting the development of fertile pollen grains in such conditions. The current understanding of the adaptation of such bracts to alpine environments mainly focuses on the phenotypic and physiological changes while the genetic basis is very limited. By sequencing the upper bract and the lower rosulate leaf from the same *R. nobile* stem, we identified candidate genes that may be involved in alpine adaption of the translucent bract in “glasshouse” plants and illustrated the changes in gene expression underlying the adaptive and complex evolution of the bracts phenotype.

**Results:**

A total of 174.2 million paired-end reads from each transcriptome were assembled into 25,249 unigenes. By comparing the gene expression profiles, we identified 1,063 and 786 genes up-regulated respectively in the upper bract and the lower leaf. Functional enrichment analyses of these genes recovered a number of differential important pathways, including flavonoid biosynthesis, mismatch repair and photosynthesis related pathways. These pathways are mainly involved in three types of functions: 9 genes in the UV protective process, 9 mismatch repair related genes and 88 genes associated with photosynthesis.

**Conclusions:**

This study provides the first comprehensive dataset characterizing *Rheum nobile* gene expression at the transcriptomic scale, and provides novel insights into the gene expression profiles associated with the adaptation of the “glasshouse” plant bracts. The dataset will be served as a public genetic resources for further functional and evolutionary studies of “glasshouse” plants.

## Background

One of the major goals of evolutionary biology is to explain the genetic basis of phenotypic adaptation [1]. Many examples of adaptive phenotypic change have been shown to be due to changes in protein coding sequence [2]. However, there is a growing body of work showing that in some cases where gene sequence is functionally conserved, gene regulation modifications can cause the major phenotypic differences that underlie adaptive changes. For example, floral color in petunia [3], fruit size in tomato [4], kernel color in maize [5], and inflorescence architecture in rice [6], have all been shown to be the result of gene expression changes rather than changes in protein structure. These studies of model organisms represent compelling evidence for the role of gene regulation in phenotypic evolution. However, most phenotypes are far more complex and controlled by hundreds of genes [7]. Previous studies have focused on a single or a few candidate genes, which limited our understanding of the molecular basis of adaptation changes in gene expression, and lacked sufficient power to identify the suites of genes and regulatory loci underlying adaptive traits. New advances in high-throughput sequencing technology made it possible to scan whole transcriptomes for all loci that have experienced changes in gene expression.

**Figure 1:**
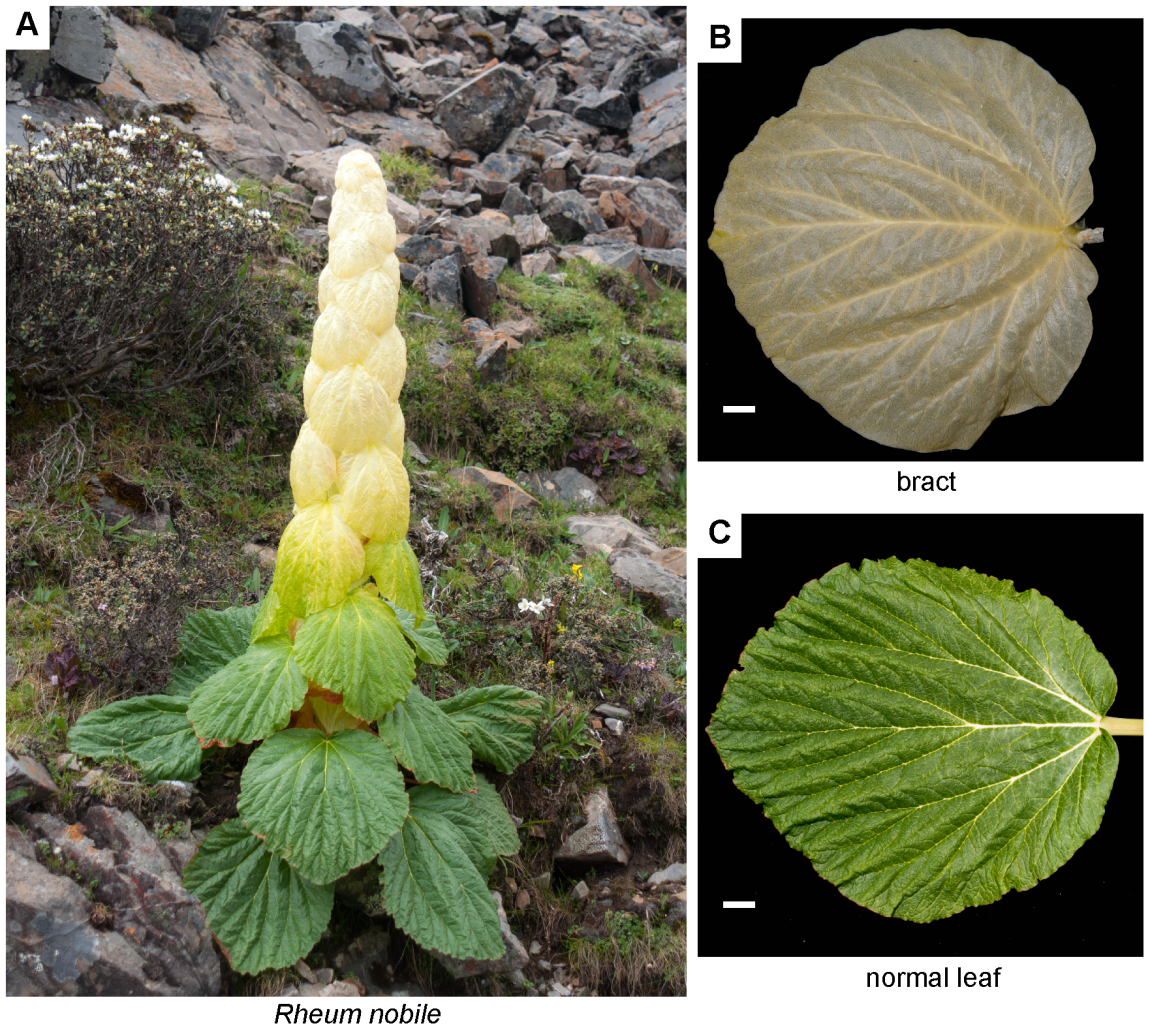
Morphological divergence of *Rheum nobile*. (A) An individual of *R. nobile*. (B) Bract. (C) Normal leaf. Bars 1 cm.

Alpine environments are usually characterized by several features such as low atmospheric pressure, low air temperature, high irradiance, strong winds and diurnal environmental fluctuations [8]–[10]. To cope with the abiotic stress of alpine environments, plants in alpine conditions have developed a variety of phenotypes [11]. One of these is “glasshouse” plants, characterized by large and showy translucent bracts concealing the inflorescence [12], [13]. *Rheum nobile* Hook. f. and Thomson (Polygonaceae), which is endemic to the alpine zones of the eastern Himalayas between 4000 and 4800 m a.s.l., has been chosen as a model species for investigating alpine adaptation of “glasshouse” plants [14]–[19]. It produces the large rosulate bracts and grows to a height of about 1.5 m (Figure 1) [20]. Experiments about their phenotypic and physiological characters indicated that their specialized bracts could enhance reproductive success during flowering and seed development in alpine conditions [10], [12], [14], [20]–[22]. The large translucent bracts of *R. nobile* are highly adapted to the environmental conditions of this region; they have a multiple epidermis structure where the cells are highly pigmented, and selectively block UV radiation while letting almost all visible light through [20], [23]. Thus, the developing flowers and the apical meristems are protected from the intense radiation found in alpine conditions. The bracts also protect the buds against wind and rain [14], [24] while trapping heat (hence “glasshouse”) and thereby promoting development of fertile pollen grains [12]. Therefore, the *R. nobile* provide an excellent model system to study how “glasshouse” species are adapted to alpine environments. Molecular processes and differential expression analysis have been studied using cDNA-AFLP gene expression approaches [14]. However, cDNA-AFLP approach has a high chance of false positives. This is because the fragment is not directly associated with a gene and a single band may represent more than one cDNA. In addition, this technique is limited by primers specific to the adapter and restriction site sequence. It can only detect and annotate a few differential expression fragment which is far from complete. Despite great progresses toward understanding phenotypic and physiological adaptations of bracts in alpine habitats, molecular basis remains largely unexplored due to the lack of genetic resources of any *Rheum* species (only 110 ESTs for *R. nobile*, 898 ones for the total genus *Rheum* in NCBI up to July 26, 2014). Novel, high-throughput, deep-sequencing technologies are making an impact on genomic research by providing new strategies to analyze the functional complexity of transcriptomes [25]. The RNA-seq approach [26] produces millions of short cDNA reads that are mapped to a reference genome to obtain a genome-scale transcriptional map, which consists of the transcriptional structure and the expression level for each gene.

In this study, we carried out the first global analysis of the *R. nobile* transcriptome using an Illumina RNA-seq method. We respectively sampled an upper bract and a normal leaf from the same *R. nobile* stem in two individuals as replicates. By comparing their transcriptomes, we found numerous interesting genes showing differential expressions between the upper bract and the lower leaf. The functional and phenotypic outcomes of these candidate genes are further inferred by annotation and enrichment analyses. We used this genome wide information to (i) define and annotate the transcriptome of *R. nobile* in order to provide further genetic resource for functional analysis, (ii) discover functional genes involved in the “glasshouse” plant adaptation to the alpine environment, and (iii) understand the relationship between gene expression and complex phenotype evolution.

## Results and Discussion

### Paired-end sequencing and *de novo* assembly

**Figure 2:**
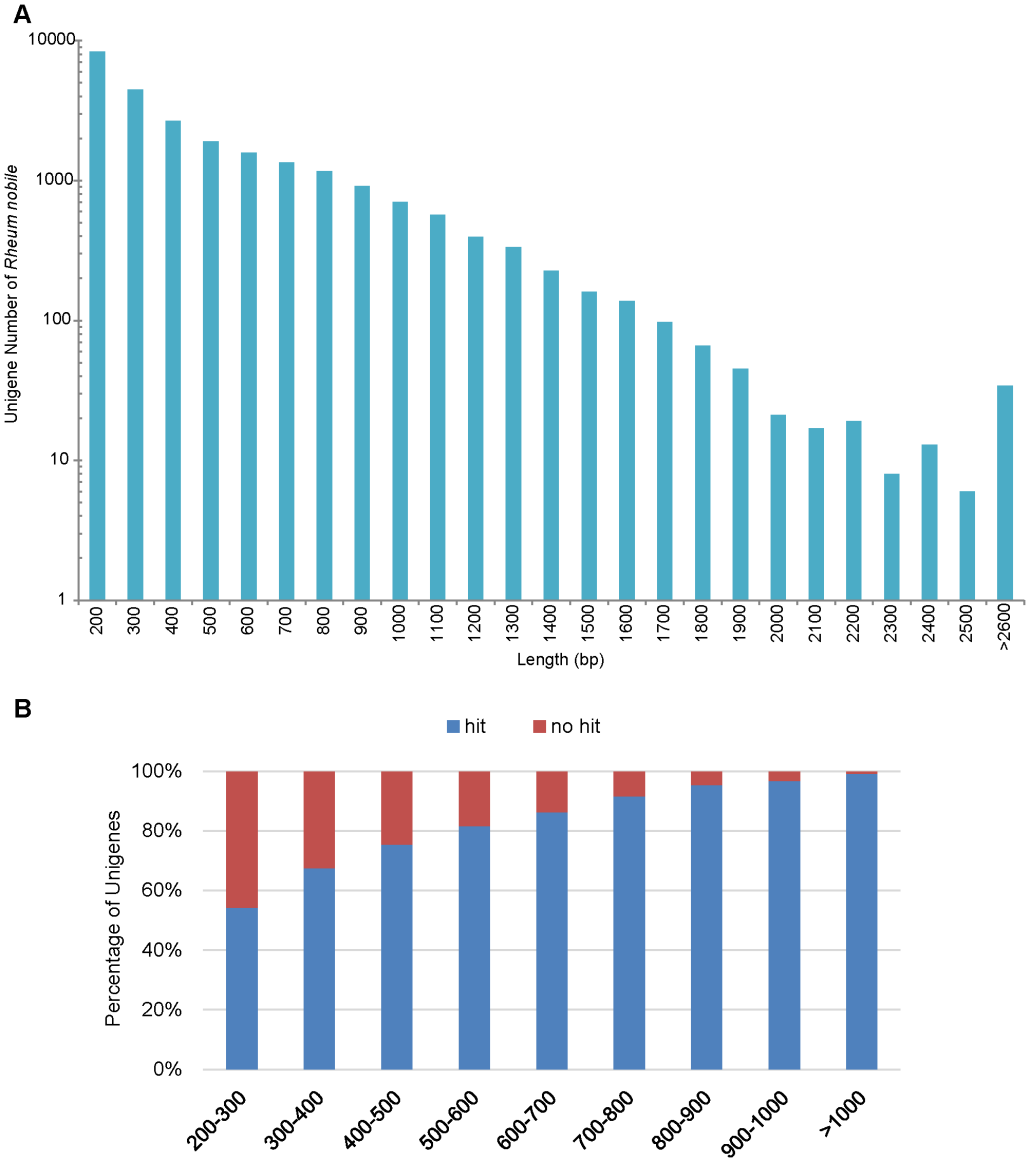
Length distribution of *Rheum nobile* unigenes and length distribution of unigenes with or without blast hits. (A) Frequency distribution of unigenes by size. (B) Comparison of unigene length with or without blast hits.

To explore the characterization of *R. nobile*, four cDNA libraries were constructed from two types of leaves (an upper translucent bract and a lower rosulate leaf, from two healthy individuals as replicates). A total of 174.2 million paired-end filtered reads were generated by Illumina sequencing after removing the adaptor and low quality reads. *De novo* assembly of filtered reads was performed by paired-end joining and gap-filling using Trinity software [27]. In total, four raw assemblies were obtained and were further merged by integrating sequence overlaps and eliminating redundancies. We obtained the final set of assemblies into 25,424 unigenes. The average length of these unigenes was 536 bp with the N50 equaling 691 bp (Table 1). The frequency distribution of unigenes by size is shown in Figure 2A. In order to exclude possible non-coding RNA, untranslated regions, and microbial sequences, we used a BLASTN algorithm to compare our unigenes to various databases (listed in Materials and Methods). Finally, we generated a total of 25,249 high-quality unigenes with 175 possibly polluted sequences excluded. We assessed the quality of the assembly by aligning the 110 ESTs for *R. nobile* downloaded from NCBI onto the unigenes we obtained. 90 of 110 (81.82%) ESTs have a blast hit with a cut-off E-value of 10^−5^. These high-quality unigenes (DatasetS1) were used for later function and differential expression analysis in this study.

**Table 1:** Summary of transcriptome assembly for *Rheum nobile*.

	Upper bracts of T0	Lower leaves of T0	Upper bracts of T1	Lower leaves of T1
Number of reads	25,625,720	24,799,850	62,769,940	60,972,084
Total nucleotides (bp)	1,883,490,420	1,822,788,975	6,276,994,000	6,097,208,400
Read length (bp)	72+75	72+75	100+100	100+100
Number of unigenes	25,424			
Maximum unigene length	8,188			
Minimum unigene length	201			
Average length of unigenes (bp)	536			
N50 size of unigenes (bp)	691			

### Functional annotation of the *R. nobile* transcriptome

For annotation, all the high-quality unigene sequences from final assembly were searched against nr, Swiss-prot, COG and KEGG databases using BLASTX [28] with a cut-off E-value of 10^−5^. A total of 18,524 out of 25,249 unigenes (73.37%) showed significant similarity to known proteins (Table 2). For unigenes with length ≥300 bp, 82.83% of them had BLASTX hits; and for unigenes with length ≥600 bp, this ratio was 94.35%. These results suggested that most of these unigenes were protein-encoding transcripts. However, 6,725 unigenes (26.63% in total; 78.49% of unigenes <400 bp, and 0.37% of unigenes>1,000 bp) could not be matched to known genes (Figure 2B), suggesting that longer unigenes were more likely to have BLAST matches in the protein databases. These unigenes longer than 400 bp and without hits (1443, 5.72%) had a high chance of putative novel transcribed sequences or species-specific sequences (Table S1).

**Table 2:** Summary of functional annotation of *Rheum nobile* total unigenes.

Database	25,249 total unigenes	
	Number of hits (e-value = 1e-5)	Percentage of total unigenes
nr	18,501	73.27%
Swiss-Prot	13,161	52.12%
GO	15,135	59.94%
COG	4,560	18.06%
KEGG	2,677	10.60%
Total	18,524	73.37%

**Figure 3:**
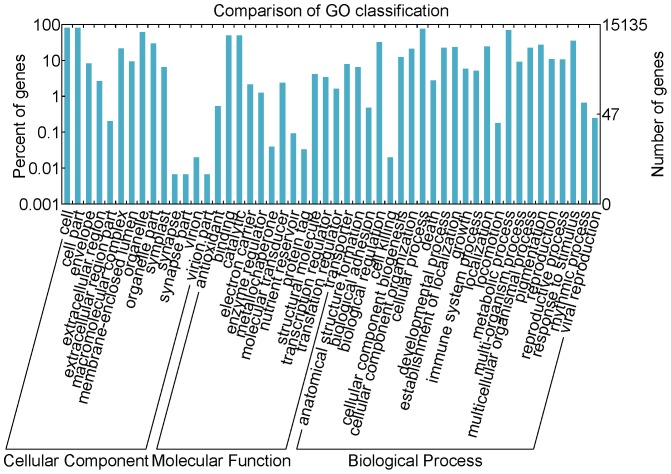
Gene Ontology classifications of *Rheum nobile*. Unigenes were aligned to the GO database, and 15,135 unigenes were assigned to at least one GO term. All the unigenes were grouped into three major functional categories, namely cellular component, molecular function and biological process. The right y-axis indicates the number of unigenes in a category. The left y-axis indicates the percentage of a specific category of unigenes in that main category.

To determine the possible functions of assembled *R. nobile* unigenes, Gene Ontology (GO) assignments were used to classify the unigene sequences based on the nr annotation. GO is an international standardized gene functional classification system which offers a dynamic-updated controlled vocabulary and a strictly defined concept to comprehensively describe properties of genes and their products in any organism [29], [30]. There were 18,501 unigenes annotated in nr, among which 15,135 unigenes could be categorized into functional groups under the “Cellular component”, “Molecular function” and “Biological process” divisions (Figure 3). The GO annotation assignment determined that the genes expressed in this study encode diverse structural, regulatory and metabolic proteins. Among the annotated unigenes, GO terms related to basic cell functions were the most abundant. Briefly, for the cellular component group, genes involved in “cell” (12149, 80.3%, GO: 0005623) and “cell part” (12149, 80.3%, GO: 0044464) were highly represented, followed by “organelle” (9323, 61.6%, GO: 0043226). For molecular function group, “binding” (7497, 49.5%, GO: 0005488) and “catalytic” (7414, 49.0%, GO: 0003824) were the most represented categories. For the biological process group, “cellular process” (11480, 75.9%, GO: 0009987) and “metabolic process” (10555, 69.7%, GO: 0008152) were the most represented categories, but it was noteworthy that “response to stimulus” (GO: 0050896) and “pigmentation” (GO: 0043473) were also highly represented, with 5,307 and 4106 unigenes involved, respectively. Unigenes with GO terms associated with reproduction were well represented, such as “reproduction” (GO: 0000003) and “reproductive process” (GO: 0022414).

**Figure 4:**
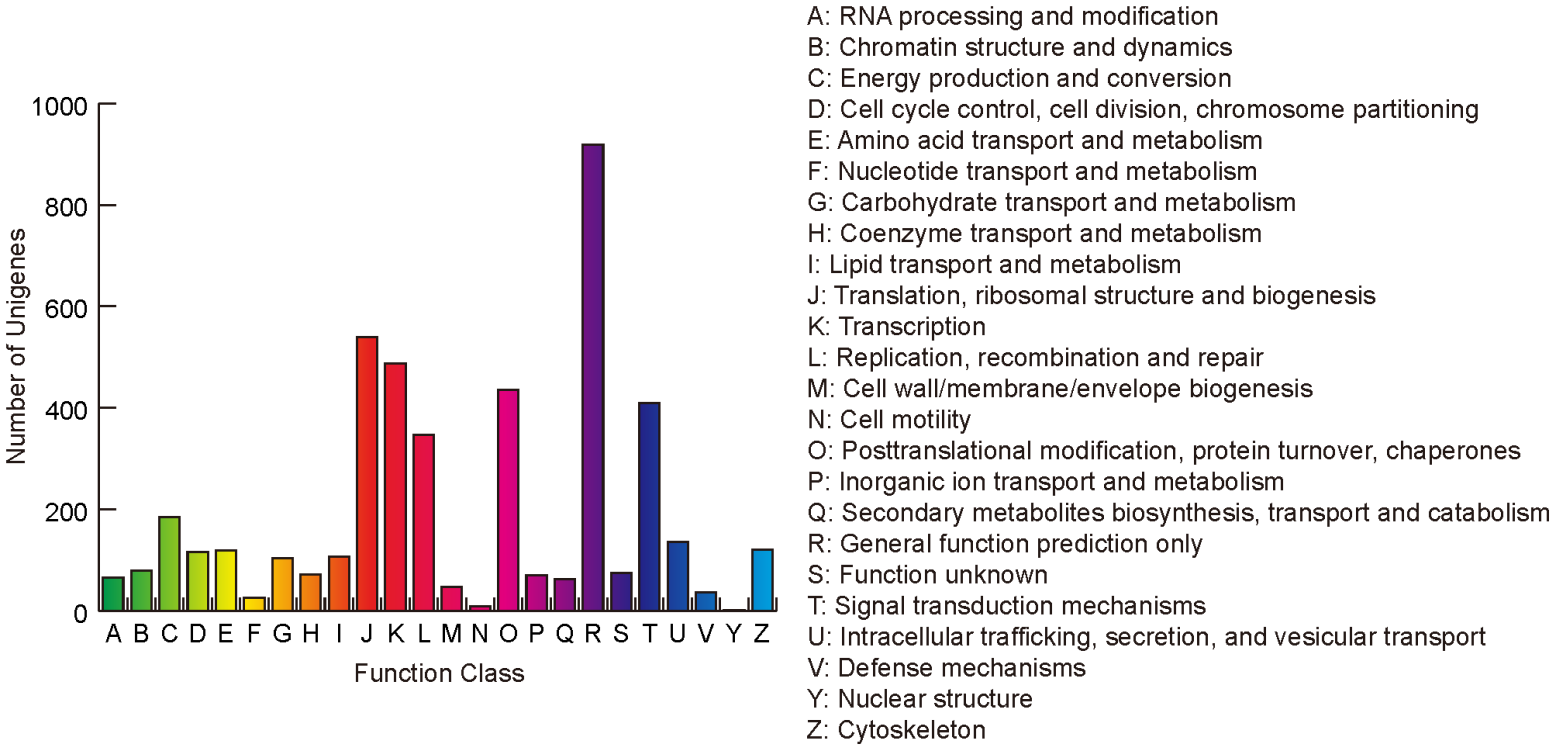
COG function classification of the *Rheum nobile* transcriptome. In total, 4,560 sequences out of 25,249 unigenes were grouped into 24 COG classifications.

To further evaluate the function of the assembled unigenes, we searched the annotated sequences for genes involved in Clusters of Orthologous Groups (COG). COG is a database where orthologous gene products were classified. Every protein in COG is assumed to be evolved from an ancestor protein, and the whole database is built on coding proteins with complete genome as well as system evolution relationships of bacteria, algae and eukaryotes [31]. Possible functions of 4,560 unigenes were classified and subdivided into 24 COG categories (Figure 4). Among the 24 categories, the cluster for ‘general function prediction’ (919, 20.2% of the matched unigenes) represented the largest group, followed by ‘translation, ribosomal structure and biogenesis’ (539, 11.8%), ‘transcription’ (487, 10.7%), ‘posttranslational modification, protein turnover, chaperones’ (436, 9.6%), ‘signal transduction mechanisms’ (410, 9.0%), ‘replication, recombination and repair’ (346, 7.6%). Only a few unigenes were assigned to ‘nuclear structure’ and ‘cell motility’ (1 and 8, respectively).

In addition, Kyoto Encyclopedia of Genes and Genomes (KEGG) pathway analysis was performed on unigenes generated in the present study, in order to further learn the biological functions of detected genes. KEGG is a pathway-related database resource that integrates genomic, chemical and systemic functional information [32], [33]. It contains systematic analysis of inner-cell metabolic pathways and functions of gene products, which aid in studying the complex biological behaviors of genes [34]. In total, 2,677 unigenes were assigned to 233 KEGG pathways (Table 3). These predicted pathways represented the majority of plant metabolism, genetic information processing, environmental information processing, cellular processes and organismal systems. Briefly, of these sequences with KEGG annotation, 1,205 (45.0%) were classified into the metabolism, including majority sub-groups of energy metabolism (355, 13.3%), carbohydrate metabolism (312, 11.7%) and amino acid metabolism (229, 8.6%). Sequences grouped into the genetic information processing, accounted for 1,158 (43.3%), including translation (571, 21.3%), folding, sorting and degradation (372, 13.9%), transcription (214, 8.0%) and replication and repair (66, 2.5%). Environmental information processing, cellular processes and organismal systems groups contained 230 (8.6%), 327 (12.2%) and 311 (11.6%) KEGG annotated sequences, respectively.

**Table 3:** KEGG biochemical mappings for *Rheum nobile*.

KEGG categories represented	Unique sequences (Number of KO)
**Metabolism**	**1,205(791)**
Amino Acid Metabolism	229(158)
Biosynthesis of Other Secondary Metabolites	60(40)
Carbohydrate Metabolism	312(172)
Energy Metabolism	355(198)
Glycan Biosynthesis and Metabolism	56(47)
Lipid Metabolism	150(103)
Metabolism of Cofactors and Vitamins	137(102)
Metabolism of Other Amino Acids	84(45)
Metabolism of Terpenoids and Polyketides	82(61)
Nucleotide Metabolism	119(84)
Xenobiotics Biodegradation and Metabolism	44(26)
**Genetic Information Processing**	**1,158(636)**
Folding, Sorting and Degradation	372(212)
Replication and Repair	66(53)
Transcription	214(130)
Translation	571(277)
**Environmental Information Processing**	**230(115)**
Membrane Transport	17(8)
Signal Transduction	211(106)
Signaling Molecules and Interaction	2(1)
**Cellular Processes**	**327(184)**
Cell Communication	23(14)
Cell Growth and Death	99(59)
Cell Motility	24(14)
Transport and Catabolism	210(114)
**Organismal Systems**	**311(159)**
Circulatory System	41(18)
Development	13(5)
Digestive System	45(18)
Endocrine System	68(34)
Environmental Adaptation	77(35)
Excretory System	48(24)
Immune System	68(39)
Nervous System	101(43)
Sensory System	17(3)
**Total**	**2,677(1,587)**

### Differentially expressed genes (DEGs) between the upper bract and the lower leaf of *R. nobile*

We performed expression level analyses to calculate fold change between upper bracts and lower leaves of *R. nobile*. In order to calculate the unigene expression levels, the RPKM value was measured by mapping reads of each library to *de novo* assembled unigene sequences using Bowtie2 software [35]. The RPKM value is able to eliminate the influence of different gene length and sequencing level on the calculation of gene expression [26]. The R package edgeR [36] was used to estimated reads counts of each gene in each sample and *p*-values were generated. False discovery rate (FDR) [37] was used to justify the *p*-values. We singled out the DEGs between upper bracts and lower leaves for their expression profiles: (i) the fold change in gene expression level ≥2 in both replicates and (ii) the FDR significance score ≤0.001 [37], [38]. In this study, DEGs with higher expression levels in bract samples when compared with normal leaf samples were denoted as “up-regulated”, while those with lower expression levels in the bract samples were denoted as “down-regulated”. Under these criteria, 1,849 out of 25,249 unigenes were identified to be differentially expressed between the upper bract and the lower leaf: 1,063 up-regulated and 786 down-regulated (Table S2). Several genes identified by a previously published gene expression study using cDNA-AFLP [14] were also found by RNA-seq. Although the total number of differentially expressed genes was different between the studies (323 in cDNA-AFLP and 1,849 in RNA-seq), similar transcripts were involved in photosynthesis, stress and defense response, DNA replication and metabolism, including photosystem II 22 kDa protein, F-type H+-transporting ATPase subunit gamma, photosystem I subunit II, photosystem I subunit III, flavonols synthase and glutamine synthetase. Besides the similar transcripts found in both studies, our RNA-seq data also discovered transcripts related to mismatch repair and transcriptional regulation.

### qRT-PCR validation of RNA-seq data

Twenty DEGs were selected to demonstrate the RNA-seq results using qRT-PCR. Specific primers were designed by Primer 3 and showed in Table S3. The qRT-PCR data showed the similar trends with RNA-seq data (Figure S1, S2), but bias in the degree of differential expression between the two data sets. In general, qRT-PCR data depicted up/down regulation patterns of DEGs were consistent with Illumina sequencing results, suggesting that Illumina data are relatively reliable.

### Functional enrichment analysis of DEGs

To gain insights the functional significance of the two different leaves (the upper bract and the lower leaf), we performed Gene Ontology (GO) enrichment analysis. Among the 1,849 DEGs, 1,512 (840 up-regulated and 672 down-regulated) were annotated to GO categories. The hypergeometric test was used to map DEGs to terms in the Gene Ontology database, looking for terms that were significantly enriched compared with the genome background. 37 GO terms had a significantly enrichment among the DEGs (Table S4).

Several up-regulated genes in the bract were associated with cellular component organization (GO: 0009664, GO: 0006334, GO: 0005794, GO: 0005730), which reflected a special cellular structure of the bracts. Previous studies about leaf anatomy of *R. nobile* found that the mesophyll of the bracts has 2–3 layers of cells which are not differentiated into palisade and spongy parenchyma [20]. These cells have few chloroplasts and the uppermost layer of the mesophyll acts as a multiple epidermis. Additionally, the up-regulated genes represented the terms associated with nucleotide metabolic process (GO: 0009220, GO: 0000462, GO: 0006220), RNA methylation (GO: 0001510) and regulation of translation elongation (GO: 0006414), may indicate their important roles for post-transcriptional regulation in bracts. These up-regulated genes coincided with previous studies [14], [20], [24], and could be the candidate genes which play major role in mesophyll structure specialization and metabolism regulation process in bract.

For down-regulated genes, all of these terms were highly correlated with photosynthesis, such as photosynthetic electron transport chain (GO: 0009767), chloroplast photosystem II (GO: 0030095), thylakoid membrane organization (GO: 0010027) and stomatal complex morphogenesis (GO: 0010103). Previous studies found that the chloroplasts from bracts were smaller than those from normal leaves and had a smaller average number of discs per grana stack, and the content of chlorophyll a, chlorophyll b and carotenoids was dramatically reduced in bracts [14]. Leaf is the important organ for photosynthetic reactions that plants depend on to finish accumulation of photosynthetic products, which are impacted greatly by physiological structures. These down-regulated genes were consistent with phenotypic characters of *R. nobile*, i.e. that the upper leaves have become large translucent bracts designed to transmit rather than absorb visible light. Our finding suggests that the bracts have altered their foliar photosynthetic functions during adaptation to alpine environments.

### Pathway enrichment analysis of DEGs

To further investigate the biochemical pathways of these DEGs, we mapped these genes to terms in KEGG database and compared this with the whole transcriptome background. Of the 1,849 DEGs, 594 (428 up-regulated and 166 down-regulated) genes had a KO ID and could be categorized into 176 pathways. Of these, seven and six pathways were significantly (*p*-value ≤0.05) overrepresented in upper bracts and lower leaves respectively (Table S5). It is noteworthy that pathways closely related to UV stress response including ‘Flavonoid biosynthesis’, ‘Mismatch repair’ and ‘DNA replication’ were significant at upper bracts; and four photosynthesis related pathways were overrepresented in lower leaves, i.e. ‘Photosynthesis’, ‘Photosynthesis-antenna proteins’, ‘Carbon fixation in photosynthetic organisms’ and ‘Carotenoid biosynthesis’, indicating weaken photosynthesis of the bracts. We will discuss the functional significance one by one in detail below.

**Figure 5:**
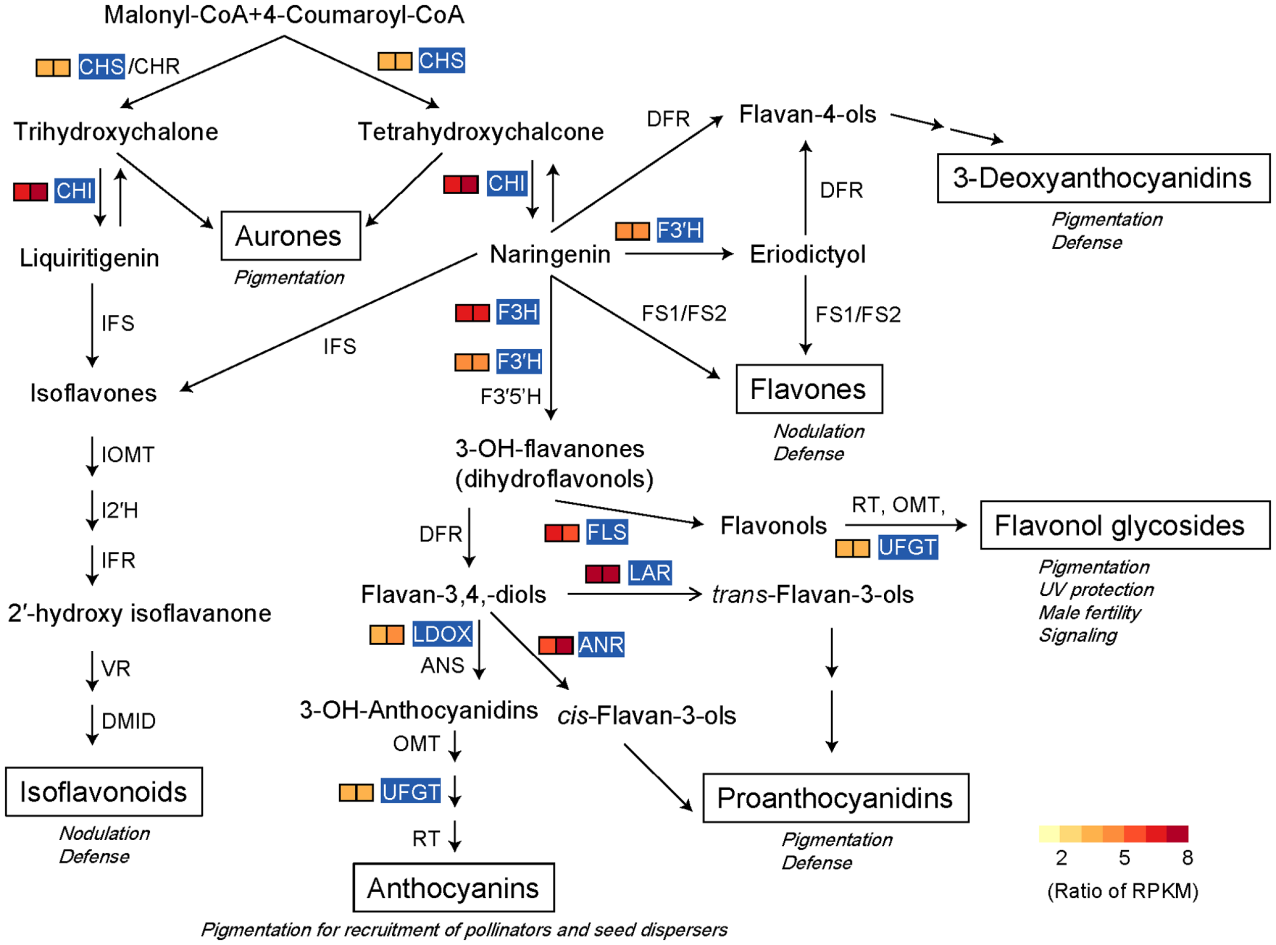
Schematic of the flavonoid biosynthetic pathway. ANR, anthocyanidin reductase; ANS, anthocyanidin synthase; CHR, chalcone reductase; CHS, chalcone synthase; CHI, chalcone isomerase; DFR, dihydroflavonol 4-reductase; DMID, 7, 2′-dihydroxy, 4′-methoxyisoflavanol dehydratase; FLS, flavonol synthase; F3H, flavanone 3-hydroxylase; F3′H, flavonoid 3′ hydroxylase; F3′5′H, flavonoid 3′5′ hydroxylase; FS1/FS2, flavone synthase; I2′H, isoflavone 2′-hydroxylase; IFR, isoflavone reductase; IFS, isoflavone synthase; IOMT, isoflavone O-methyltransferase; LAR, leucoanthocyanidin reductase; LDOX, leucoanthocyanidin dioxygenase; OMT, O-methyltransferase; RT, rhamnosyl transferase; UFGT, UDPG flavonoid glucosyl transferase; VR, vestitone reductase. The names of the major classes of flavonoid endproducts are boxed. Some of the known functions of the compounds in each class are indicated in italics. Genes annotated in significantly enriched KEGG pathway in differentially expressed genes are highlighted in blue. Individual T0 (left) and T1 (right) are indicated in 2-box strings. Heat maps were drawn using ratio of RPKM (upper bract/lower leaf) values. Red indicates high RPKM value, green indicates low RPKM value. Adapted from [64].

Flavonoids are ubiquitous plant secondary metabolites that can be divided into subgroups including chalcones, flavones, flavonols, flavandiols, anthocyanins, condensed tannins (or proanthocyanidins) and aurones. Flavonoids have substantial UV absorption properties and most of the attenuation of UV radiation is the result of absorption and scattering within epidermal tissue [39]–[41]. It has been shown that the uppermost layer of the mesophyll in *R. nobile* leaves acts as a multiple epidermis, and its cells are highly pigmented [20]. There are likely to be many flavonoid pigments dissolved in these cells to screen UV radiation. Additionally, flavonoids are known to be the major red, blue and purple pigments in plants [42]. With the accumulation of flavonoids, showy bracts are an effective way to attract pollinators during flowering [43]. On the basis of our annotation of *R. nobile* transcriptome, we identified 9 putative genes up-regulated in bracts involved in the flavonoid pathway (Table S6; Figure 5). Chalcone synthase (CHS, EC: 2.3.1.74), which catalyzes the stepwise condensation of three acetate residues from malonyl-CoA to yield naringenin chalcones, is a key enzyme in the synthesis of flavonoids [44]. Our results showed that a possible *Arabidopsis thaliana* CHS (also known as TT4) homologue gene (comp84717_c0_seq2) was up-regulated in bracts. Another enzyme that plays a key role in flavonoid biosynthesis, chalcone isomerase (CHI, EC:5.5.1.6), converts naringenin chalcones into naringenin to form the primary C15 flavonoid skeleton [45]. Our results clearly indicated that one transcript (comp88433_c0_seq1), putatively homologous to *Arabidopsis thaliana* CHI (also known as TT5) gene, was also up-regulated in bracts. From these central intermediates, the flavonoid biosynthesis pathway diverges into several side branches, each resulting in a different class of flavonoids. We also observed seven putative genes (F3H, FLS, F3′H, LDOX, ANR, LAR and UFGT) that likely catalyze succedent flavonoid compounds in bracts, indicating the overrepresented of the flavonoid biosynthesis pathway in bracts.

**Figure 6:**
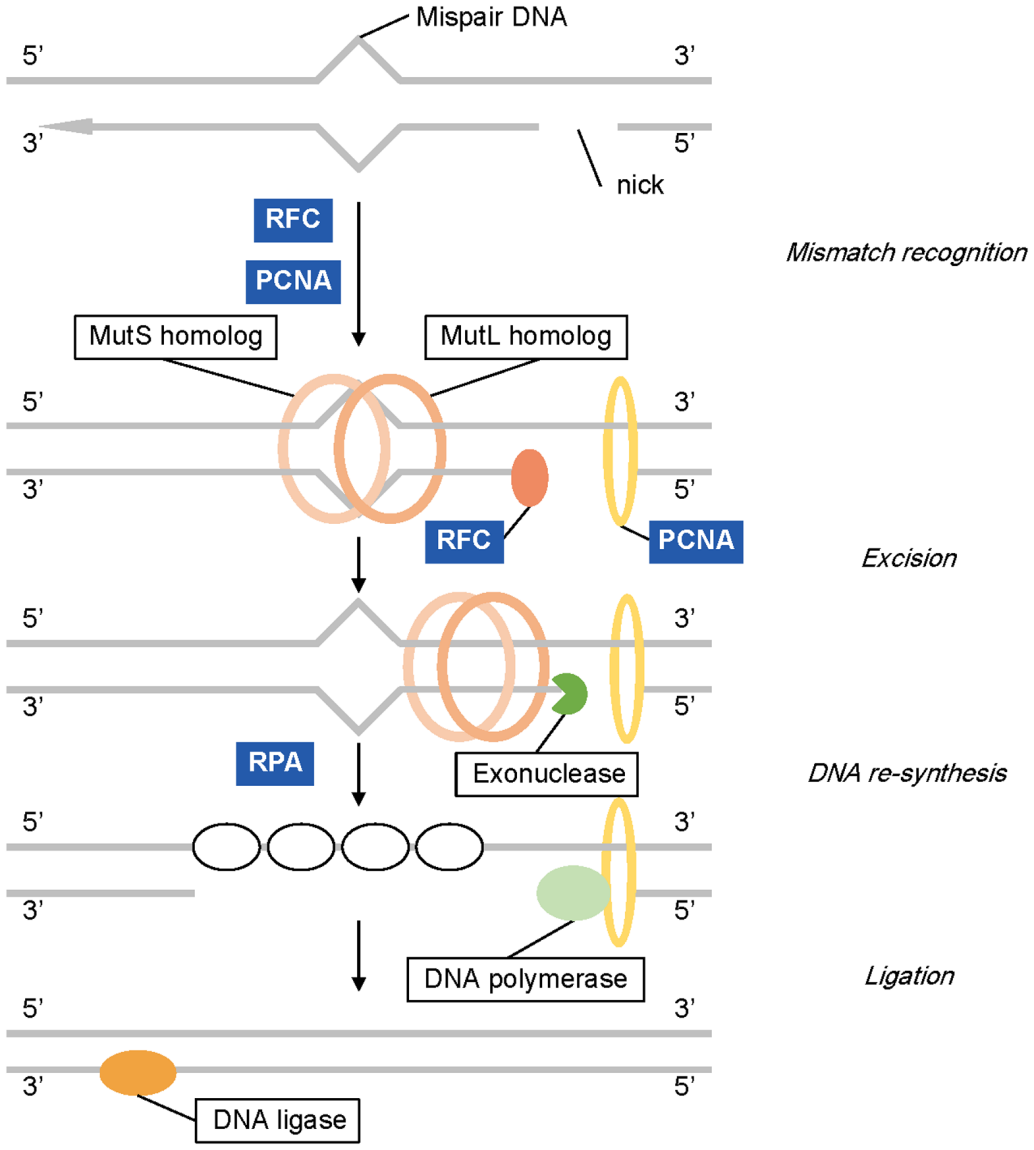
The mismatch repair pathway. Genes up-regulated significantly (P-value ≤0.05) enriched in mismatch repair pathway are highlighted in blue.

The maintenance of DNA integrity is vital for the viability of cells and the health of organisms [46]. DNA mismatch repair prevents such mutations that probably disturb DNA integrity in dividing cells and therefore maintains genomic stability [47], [48]. The “glasshouse” bract increases flower and fruit temperature on sunny days, greatly decreases the intensity of UV radiation reaching flowers and fruits and prevents pollen grains being washed away by rain [24]. Artificial removal of translucent bracts might lead to abnormal microsporogenesis and few reproductive grains in *R. nobile* [12]. In this study, we found 9 mismatch repair related genes which were up-regulated in the upper bract compared with the lower leaf (Figure 6, Table S6). Their homologues were found to play an important role in mismatch repair at the step of mismatch recognition or initiation and elongation of DNA repair [39], [49]–[53].

To investigate photosynthesis related pathways, the expression patterns of the transcripts encoding key proteins were analyzed. The light-harvesting chlorophyll protein complex (LHC) in the photosynthetic membrane performs specialized functions which result in the biochemical fixation of the solar energy [54]. A total of 19 genes involved in the LHC photosynthesis-antenna proteins pathway were down-regulated in bracts (Table S6). Additionally, both photosystem II and I composed of multisubunit complexes in the thylakoid membranes are vital for photosynthesis in higher plants [55]. Among significantly down-regulated genes in the bract, we detected a total of seventeen genes encoding photosystem II complex, ten genes encoding photosystem I subunit, six genes encoding ATPase subunit, two genes encoding cytochrome b6-f complex, two genes encoding ferredoxin, one gene encoding plastocyanin, nine genes involved in carotenoid biosynthesis, and twenty-two gene involved in carbon fixation in photosynthetic organisms (Table S6). Obviously, these results, in accordance with previous observations [12], [14], suggested the strong weakening of photosynthesis functions in the bract.

## Conclusions

Various reports have provided evidence that *R. nobile* is morphologically highly adapted to the alpine environment and its bracts produce a “glasshouse” effect for the inflorescence, thus heating it and promoting development of fertile pollen grains. Our study provides a first transcriptomic analysis of *R. nobile* adaptation to alpine conditions and increases the number of annotated *R. nobile* mRNA sequences from 110 to almost 19,000. We also demonstrated that comparing gene expression profiles by *de novo* transcriptome sequencing offers a fast and cost-effective approach to understand relationship between gene expression and complex phenotype evolution at the genomic level.

Unlike animals, higher plants, which are sessile, cannot escape from the unfriendly surroundings, but have to adapt themselves to the changing environments by modifying themselves [11]. Our results provided the molecular bases of such modifications for an alpine plant *R. nobile*. Nine genes involved in flavonoid biosynthesis, which may play important roles in UV protection and further in enhancing pollinator visitation of *R. nobile* in alpine conditions, were identified as significantly up-regulated in the bract, particularly those genes associated with chalcone synthase (CHS) and chalcone isomerase (CHI). The other up-regulated genes in the bracts further included the mismatch repair related genes, which may have ensured the functional stability of the bract with high UV radiation. However, those genes involved in photosynthesis were down-regulated in the bract. It appears that the bracts of the “glasshouse” plants have altered their function as the normal leaves, but developed as a specialized leaf to promote the development of the fertile pollen grains. Further functional analyses should illustrate how these regulatory changes lead to the phenotypic changes.

## Materials and Methods

### Ethics Statement

No specific permits were required for the described field studies. Additionally, we confirm that the field studies did not involve endangered or protected species.

### Plant Material and mRNA Sequencing

Two adult individuals (T0 and T1) of *R. nobile* with no clinical signs of disease was chosen for analysis as replicates, from its native area, the Sejila mountains, southwest Qinghai-Tibetan Plateau (29°37.081_N, 94°38.899_E), at an altitude of 4,663 m. We chose two types of leaves (the upper translucent bract and the lower rosulate leaf) for analyses with both samples were snap-frozen in liquid nitrogen and stored an −80°C for RNA extraction.

The total RNAs were isolated using the TRIzol reagent (Invitrogen, Carlsbad, CA, USA) and the RNeasy Kit (Qiagen, Hilden, Germany) approach. In addition to the A_260_/A_280_ and A_260_/A_280_ ratios, the integrity of the RNA samples was examined with an Agilent 2100 Bioanalyzer; their RIN (RNA integrity number) values ranged from 8.6 to 10.0, with no sign of degradation. RNAs with poly (A) tail were purified from total RNA by oligo (dT) magnetic beads and fragmented into short sequences. Subsequently, cDNAs were synthesized and purified with PCR extraction kit (QiaQuick). A 75-bp and/or a 100-bp paired-end sequencing protocol with insert size of 200 bp was employed using the Illumina Genome Analyzer IIx sequencing platform. Data analysis and base calling were performed by the Illumina instrument software. The entire process followed a standardized procedure and was monitored by BGI's Quality Control System.

### *De novo* Assembly by Trinity Software

The raw reads were filtered using in-house PERL script by removing adaptor sequences, empty reads, low quality sequences (i.e. those with large numbers of ‘N’s), and reads with more than 10% Q <20 bases. The filtered reads were assembled into unigenes using Trinity with default parameters, which efficiently constructs and analyses sets of *de Bruijn* graphs, fully reconstructs a large fraction of transcripts, including alternatively spliced isoforms and transcripts from recently duplicated genes [27]. We then used CD-HIT-EST [56] and CAP3 [57] to produce a final assembly by integrating sequence overlaps and eliminating redundancies.

To obtain high-quality unigene sequences for further annotation and analysis, we excluded unigene sequences which had a high similarity compared with known non-coding RNAs sequence in the Rfam [58] database. Furthermore, unigene sequences assigned to microbial (MBGD [59]), fungal, virus and bacteria (fungal, virus and bacteria sequences based on data downloaded from the NCBI database) sources were also filtered out. In addition, unigene sequences for which>50% of the bases aligned with sequences in UTRdb [60] or contained <200 non-UTR bases were excluded.

### Data deposit

The data sets of Illumina sequencing have been deposited in the NCBI Sequence Read Archive database under BioProject PRJNA194456.

### Functional annotation

We compared all high-quality unigenes against with the NCBI (National Center for Biotechnology Information) nr (non-redundant database), Swiss-Prot, COG (cluster of orthologous groups databases) and KEGG (Kyoto Encyclopedia of Genes and Genomes) using BLASTX with an E-value of 10^-5^. To annotate Gene Ontology terms describing biological processes, molecular functions and cellular components of unigenes, we used the Blast2GO [30] program based on the nr annotation. GO functional classification for all unigenes was performed using WEGO [29] (Web Gene Ontology Annotation Plot) software. COG function classification analysis of all unigenes sequences was done by in-house PERL scripts. KEGG metabolic pathway annotation was done with the KAAS [61] (KEGG Automatic Annotation Server) annotation server using the BBH (bi-directional best hit) method. The output of KEGG analysis includes KO (KEGG Orthology) assignments and KEGG pathways that are populated with the KO assignments.

### Gene expression analysis

To estimate the level of unigene transcript abundance, we count the number of reads mapped to each distinct unigene per kilobase per million (RPKM [26]) by mapping reads of each library to *de novo* assembled unigene sequences using Bowtie2 [35] software. Fold change for each unigene between upper bract and lower leaf samples were computed as the ratio of the RPKM values (0.001 was used instead of 0 if RPKM was 0). The estimated reads counts of each gene were analyzed by using the Bioconductor edgeR package [36] in the R environment (version 3.0.1). In order to discover biologically important changes in expression, the “calcNormFactors” normalization function of edgeR package was applied. This function normalizes the data by finding a set of scaling factors for the library sizes that minimizes the log-fold changes between the samples. The scale factors were computed through a trimmed mean of M-values (TMM) between samples [62]. The differentially expressed genes were detected by applying a generalized linear model likelihood ratio test, which is based on the idea of fitting negative binomial with the Cox-Reid dispersion estimates [36]. The *p*-values were generated by edgeR and the false discovery rate (FDR) was used to justify the *p*-values [37]. An absolute fold change ≥2 (fold change showed the same direction in sample T0 and T1) and a FDR significance score ≤0.001 were used as the threshold to determine differentially expressed genes (DEGs) between upper bracts and lower leaves [38].

### Validation by Quantitative real-time PCR

Methods of RNA extraction and cDNA preparation were the same as described above. Twenty genes (ten up-regulated and ten down-regulated) were selected for the confirmation of DEGs by quantitative real-time PCR (qRT-PCR) in upper bracts and lower leaves. Herein, specific primers were designed by Primer 3 (http://primer3.sourceforge.net/) and showed in Table S3. Quantification of gene expression was performed by continuously monitoring SYBR Green fluorescence. The reactions were performed in triplicate in a total volume of 10 µl. Each reaction included 5 µl of SYBR Green Master Mix (Life Technologies), 2.0 µl of direct and reverse primers, 0.5 µl of cDNA and 2.5 µl of water. Template free controls for each primer pair were included in each run. The amplification conditions were as follows: 95°C for 3 min, 40 cycles at 95°Cfor 10 s, 56°C for 15 s, and 72°C for 15 s. For a relative comparison of gene expression, we analyzed the results of the real-time PCR data with the comparative Ct method (2^-ΔΔCT^) [63], normalized to that of the housekeeping gene *Rn_actin-6*. qRT-PCR was conducted in an ABI PRISM 7500 HT (Applied Biosystems, USA).

### Gene Ontology and KEGG Orthology enrichment analyses for DEGs

GO enrichment analysis of functional significance applies a hypergeometric test to map all differentially expressed genes to terms in GO database, looking for significantly enriched GO terns in DEGs comparing to the genome background. The calculating formula is:
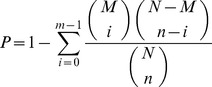



Where N is the number of all genes with GO annotation; n is the number of DEGs in N; M is the number of all genes that are annotated to the certain GO terms; m is the number of DEGs in M. We selected the *p*-value 0.05 as the threshold to determine significant enrichment of the gene sets. KEGG pathway enrichment analysis identifies significantly enriched metabolic pathways or signal transduction pathways in DEGs, comparing with the whole genome background. The calculating formula is the same as that of GO analysis.

## Supporting Information

Figure S1 qRT-PCR confirmation of RNA-seq results. qRT-PCR confirmation (left Y-axis, green bars) and RPKM (right Y-axis, purple bars) of ten up-regulated DEGs between normal leaf and bract. Relative Expression Ratios (RER) was calculated using the ΔCt method. Error bars represent standard error of means.
(TIFF)

Figure S2 qRT-PCR confirmation of RNA-seq results. qRT-PCR confirmation (left Y-axis, green bars) and RPKM (right Y-axis, purple bars) of ten down-regulated DEGs between normal leaf and bract. Relative Expression Ratios (RER) was calculated using the ΔCt method. Error bars represent standard error of means.
(TIFF)

Table S1 Table containing unigene ID and length of novel transcripts identified in this analysis.
(XLS)

Table S2 Table containing DEGs between upper bracts and lower leaves of *R. nobile*.
(XLS)

Table S3 Table containing qRT-PCR candidate and housekeeping gene primers.
(XLS)

Table S4 Table containing functional enriched GO of DEGs.
(XLS)

Table S5 Table containing enriched pathways of DEGs in KEGG database.
(XLS)

Table S6 Table containing gene list of enriched pathways in KEGG database.
(XLS)

Dataset S1 All unigene sequences obtained in this study.
(ZIP)
