# Supplementary figures and images for: Genome-Scale Transcriptome Analysis of the Alpine “Glasshouse” Plant Rheum nobile (Polygonaceae) with Special Translucent Bracts
Source: PLoS One. 2014 Oct 24;9(10):e110712. doi: 10.1371/journal.pone.0110712 (PMC4208811; doi:10.1371/journal.pone.0110712)

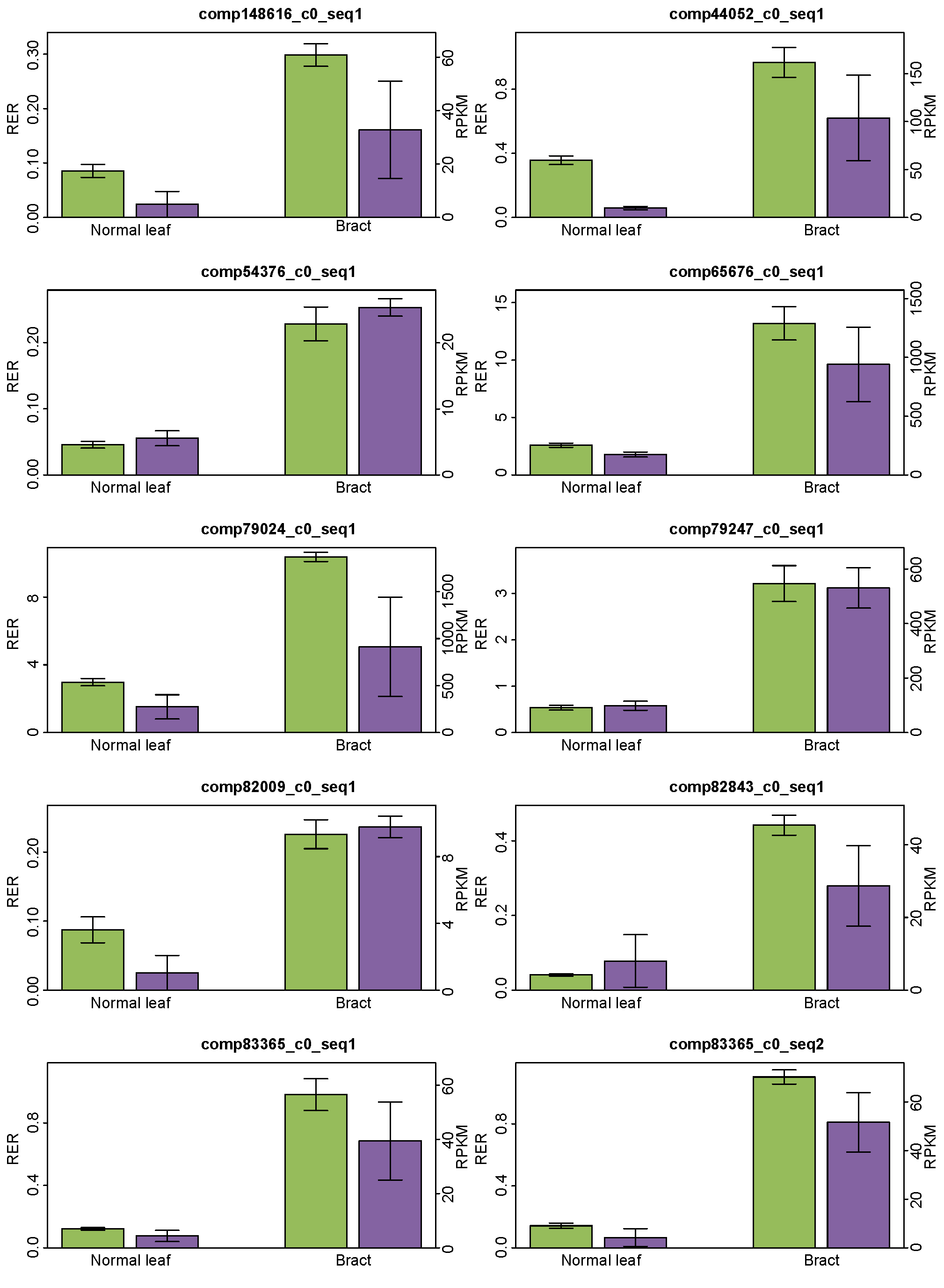

Supplement: Figure S1 — qRT-PCR confirmation of RNA-seq results. qRT-PCR confirmation (left Y-axis, green bars) and RPKM (right Y-axis, purple bars) of ten up-regulated DEGs between normal leaf and bract. Relative Expression Ratios (RER) was calculated using the ΔCt method. Error bars represent standard error of means. (TIFF) [file pone.0110712.s001.tiff]

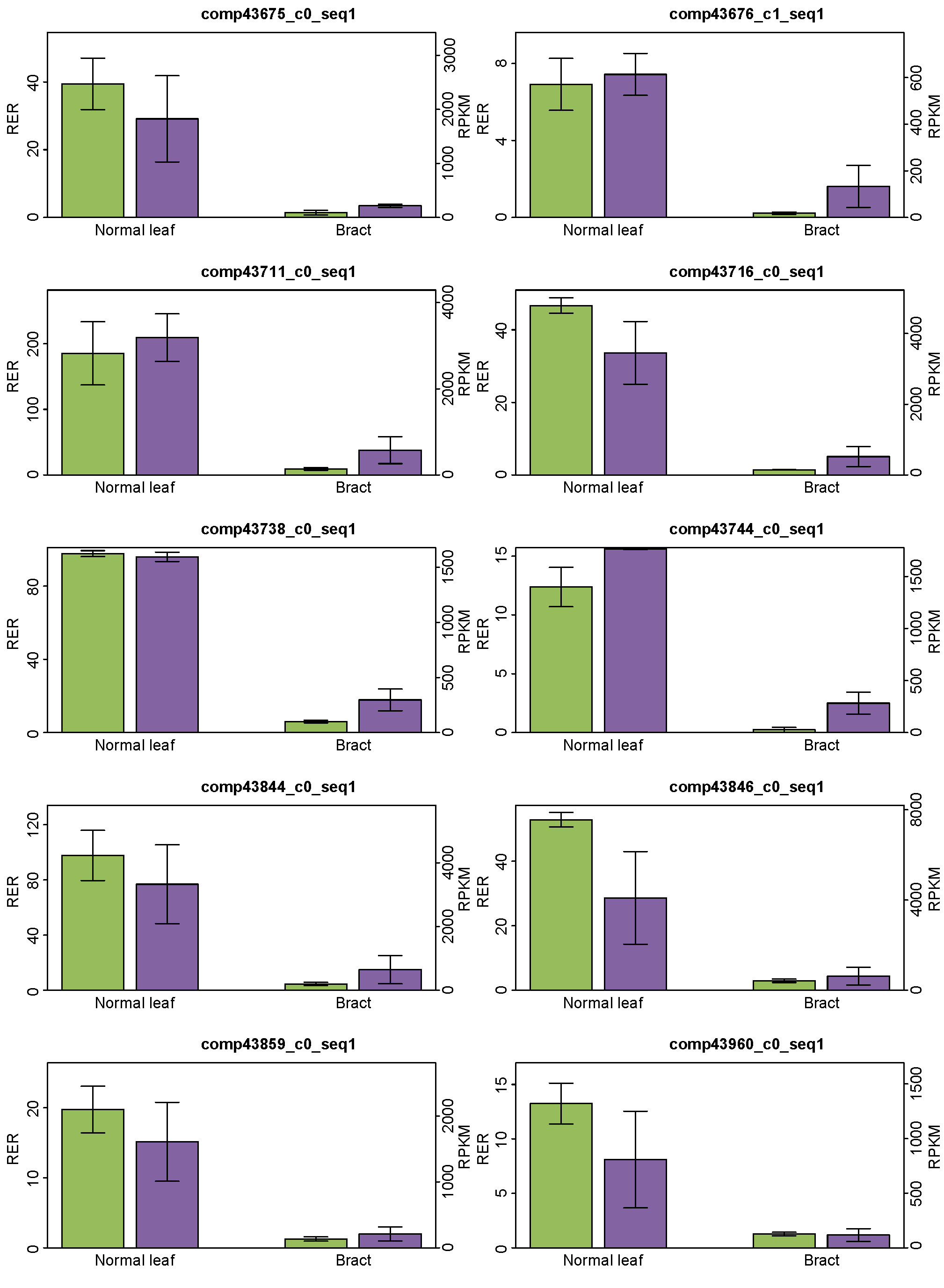

Supplement: Figure S2 — qRT-PCR confirmation of RNA-seq results. qRT-PCR confirmation (left Y-axis, green bars) and RPKM (right Y-axis, purple bars) of ten down-regulated DEGs between normal leaf and bract. Relative Expression Ratios (RER) was calculated using the ΔCt method. Error bars represent standard error of means. (TIFF) [file pone.0110712.s002.tiff]
